# Swiss Pilot Low-Dose CT Lung Cancer Screening Study: First Baseline Screening Results

**DOI:** 10.3390/jcm12185771

**Published:** 2023-09-05

**Authors:** Lisa Jungblut, Harry Etienne, Caroline Zellweger, Alessandra Matter, Miriam Patella, Thomas Frauenfelder, Isabelle Opitz

**Affiliations:** 1 Institute of Diagnostic and Interventional Radiology, University Hospital Zurich, Raemistrasse 100, CH-8091 Zurich, Switzerland; 2 Department of Thoracic Surgery, University Hospital Zurich, Raemistrasse 100, CH-8091 Zurich, Switzerland

**Keywords:** non-small cell lung cancer (NSCLC), screening, computed tomography, diagnosis

## Abstract

This pilot study conducted in Switzerland aims to assess the implementation, execution, and performance of low-dose CT lung cancer screening (LDCT-LCS). With lung cancer being the leading cause of cancer-related deaths in Switzerland, the study seeks to explore the potential impact of screening on reducing mortality rates. However, initiating a lung cancer screening program poses challenges and depends on country-specific factors. This prospective study, initiated in October 2018, enrolled participants meeting the National Lung Cancer Study criteria or a lung cancer risk above 1.5% according to the PLCOm2012 lung cancer risk-model. LDCT scans were assessed using Lung-RADS. Enrollment and follow-up are ongoing. To date, we included 112 participants, with a median age of 62 years (IQR 57–67); 42% were female. The median number of packs smoked each year was 45 (IQR 38–57), and 24% had stopped smoking before enrollment. The mean PLCOm2012 was 3.7% (±2.5%). We diagnosed lung cancer in 3.6% of participants (95%, CI:1.0–12.1%), with various stages, all treated with curative intent. The recall rate for intermediate results (Lung-RADS 3,4a) was 15%. LDCT-LCS in Switzerland, using modified inclusion criteria, is feasible. Further analysis will inform the potential implementation of a comprehensive lung cancer screening program in Switzerland.

## 1. Introduction

Lung cancer is a major public health burden. In Europe, it ranks third among the most common cancers and has the highest cancer-related death rate [1]. In Switzerland, 4500 persons are diagnosed annually with lung cancer, which is the most frequent cause of cancer-related deaths with 3200 deaths [2]. A large share of this burden would be preventable through smoking cessation or early detection of suspicious lung nodules [3]. Screening for lung cancer using low-dose computed tomography (LDCT) has been proven to be effective in detecting early stage lung cancers and has been recommended by numerous professional organizations, including the National Comprehensive Cancer Network (NCCN) and the United States Preventive Services Task Force (USPSTF) [4,5]. The NELSON trial, which is the largest randomized controlled trial (RCT) on lung cancer screening in Europe, showed that lung cancer mortality can be significantly reduced over a 10-year period by using LDCT [6].

With the publication of the results of the US National Lung Screening Trial in 2011, lung cancer mortality was reduced by 20% and all-cause mortality by 6.7% (relative risk reduction) in a clearly defined high-risk cohort [7]. In absolute numbers: 13 out of 1000 screened smokers died of lung cancer in the LDCT group vs. 17 out of 1000 in the chest X-ray group. Based on these promising data, since 2015, the costs for the screening program have been reimbursed by private as well as public health insurers in America [8]. In January 2020, Croatia became the first European country to launch a national lung cancer screening program targeting all active smokers (or those who have quit smoking in the last 15 years) between 50 and 70 years [9]. Poland has introduced a lung cancer screening program as part of its national cancer plan funded by the Ministry of Health [10,11]. The United Kingdom has also introduced regional programs; with the help of a mobile “screening truck”, a very high level of adherence has been achieved, especially in remote areas [12].

According to the last published statistics from the Federal Statistic Office, 27% of the Swiss population above 15 years old are smokers, with the 6% being heavy smokers (>20 cigarettes/day) [13]. In the Zurich Canton, the prevalence of active smokers goes up to 28.2% [14]. Nevertheless, to date, there is no official screening program in Switzerland. This may be based on obstacles like lack of country-specific data or ambiguity on the cost–benefit ratio. To ensure the sustainability of a lung cancer screening program, it would be important for the screening to be covered by compulsory health insurance (KVG) in the long term. According to Art. 12d KLV (Ordinance on Health Care Services), compulsory health care insurance covers certain medical preventive measures for the early detection of diseases in certain risk groups. Each new screening measure must be assessed for its effectiveness, appropriateness, and cost-efficiency before it is covered by compulsory health insurance. However, there is rising evidence that LDCT screening is cost-effective in Switzerland [15].

The objective of this project was to assess feasibility of introducing LDCT-LCS in Switzerland and propose characteristics for its implementation.

## 2. Materials and Methods

### 2.1. Study Design

In this prospective study, starting from October 2018, asymptomatic participants aged 55–74 years with more than 30 pack years of smoking history were enrolled at a tertiary hospital in Switzerland.

### 2.2. Inclusion Criteria

We propose the use of the already established inclusion criteria from the national lung cancer screening study [9]:

- Age from 55 to 74 years;
- Willingness and ability to undergo LDCT;
- >30 packs years;
- Current or former smoker who quit smoking ≤ 15 years ago;
- No previous diagnosis of lung cancer;
- No major medical problems;
- No CT scan in the last 18 months;
- No haemoptysis or weight loss > 7 kg in the last year.

Further, individuals with a of 1.5% probability of suffering from lung cancer within the next 6 years or higher according to PLCOm2012 risk prediction model were also eligible for LDCT lung cancer screening. The PLCOm2012 risk prediction model is based on the Prostate, Lung, Colorectal, and Ovarian Cancer Screening Trial (PLCOm2012) and incorporates 15 predictors, including medical history, sociodemographic characteristics, smoking exposure, and medical history of chronic obstructive pulmonary disease (COPD) [16].

### 2.3. Recruitment

Participants were recruited through primary care providers (PCPs), pulmonologists, but also through newspaper articles, social media, and flyers in doctors’ waiting room areas. Information about eligibility criteria as well as contact details were given. All individuals were invited for a telephone interview to check for eligibility. Further, patient information as well as smoking cessation advice and allocation to smoking cessation program were available as part of the interview. Telephone interviews were conducted by a healthcare worker (resident or medical student) employed by the radiology department.

### 2.4. Image Acquisition/Reporting

**Figure 1:**
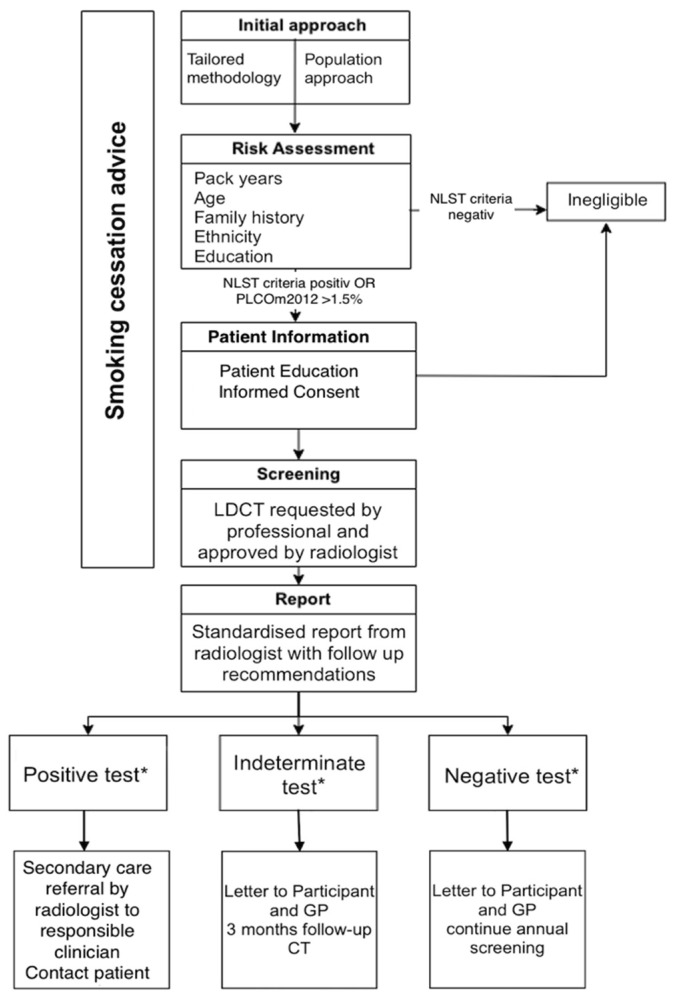
Screening pathway. GP, general practitioner. * positive test = lung-RADS 4, intermediate test = lung-RADS 3, negative test = lung-RADS 1 or 2.

LDCTs were acquired using a Siemens Somatom Force, Siemens Somatom Edge Plus and a Siemens Somatom Naeotom Alpha scanner starting from October 2019 without the administration of contrast medium. For quality control, technical standards from the ESTI (European Society of Thoracic Imaging) society were obtained [12]. Each scan was read by two radiologists independently, one of whom was board certified and specialized in thoracic radiology. In all, scan nodules were automatically detected and measured by the software’s built-in matching algorithm (Siemens SyngoVia MM oncology lung computer-aided detection [CAD]) and the maximum diameter was double-checked by the radiologists. Reporting was completed in a standardized way to obtain imaging parameters such as radiation dose, summary of screening findings with specific management recommendation, and additional findings. Standardized templates were used to ensure uniform reporting and guideline adherence. Nodules were classified by the Lung-RADS 1.1 reporting system [13]. Lung-RADS (Lung Imaging Reporting and Data System) is a classification for lung nodules in low-dose CT screening exams with the purpose of standardizing follow-up and management decisions. A flowchart of the screening pathway is provided in Figure 1.

### 2.5. Ethical Statement

This study respects the principles of the Declaration of Helsinki concerning human research study. This project is not subject to approval by the ethics committee of Zurich (KEK Zuständigkeitsabklärung—BASEC-Nr. Req-2017-00511).

### 2.6. Primary and Secondary Endpoint

The primary endpoint of this study was the incidence of lung cancer detection by screening. The second endpoint was the detection of indeterminate nodules, quantification of incidental findings, and consecutive recall rate.

### 2.7. Statistical Analysis

Quantitative variables were expressed as mean ± standard deviation (SD). Categorical variables were expressed as frequencies or percentages. Descriptive epidemiological summaries of data were presented with confidence intervals (CIs). For non-normally distributed continuous data, the 25th, 50th, and 75th percentiles are presented, denoting the median and interquartile range (IQR). Statistical analyses were conducted using commercially available software (IBM SPSS Statistics, release 21.0; SPSS, Chicago, IL, USA).

## 3. Results

### 3.1. Participant Cohort

A telephone interview with 150 individuals with high-risk factors was conducted. We excluded 38 participants (34 participants were not willing to sign an informed consent and 4 patients were not eligible), resulting in a final cohort of 112 participants: 65 (58%) men and 47 (42%) women. The mean age at enrollment was 62.1 (95% CI 60 to 63) years. The proportion of current smokers was 76% (*n* = 85). With regard to comorbidities, 14% (*n* = 16) of the enrolled participants had known COPD, emphysema, or bronchitis. Detailed information is shown in Table 1. Regarding the recruitment strategy, 56 (50%) of the participants became aware of the study through flyers, social media and newspapers, whereas only 28 (25%) were invited by either PCPs or other clinicians.

The majority of patients (82, 73%) came from the Zurich Canton. The median distance covered to reach the University Hospital was 16.7 km (IQR: 1.3–28), and most participants (64, 57%) chose public transport. Of the participants, 92 (82%) had an educational level beyond high school graduation; 63 (56%) were currently working, and 66 (59%) had public health insurance.

### 3.2. Radiation Dose

The mean volume CT dose index (CTDIvol) was 0.69 ± 0.18 mGy and the dose length product (DLP) was 21.04 ± 4.36 mGy*cm.

### 3.3. Lung-RADS Findings

The percentage of negative tests (Lung-RADS 1 and Lung-RADS 2, respectively) was 81% (*n* = 91), whereas the prevalence of positive LDCT results (biopsy-proven carcinomas) was 3.6% (*n* = 4). Subjects with positive results were referred to thoracic surgery for immediate assessment following the baseline scan. Intermediate results (Lung-RADS 3) were found in 13% (*n* = 14) of participants. Those patients were given an outpatient appointment at the thoracic surgery consultations and were also advised for a follow-up scan considering Lung-RADS criteria [13]. Further, not all suspected lesions were biopsied or surgically extracted. Three participants were diagnosed with Lung-RADS 4a lesions (with a malignancy rate according to Lung-RADS criteria of 5–15%) and are currently under active surveillance. Taking all lesions requiring follow-up scans into account (Lung-RADS 3 and Lung-RADS 4a) resulted in a recall rate of 15%. The results are shown in Table 2.

### 3.4. Detected Lung Cancer

**Figure 2:**
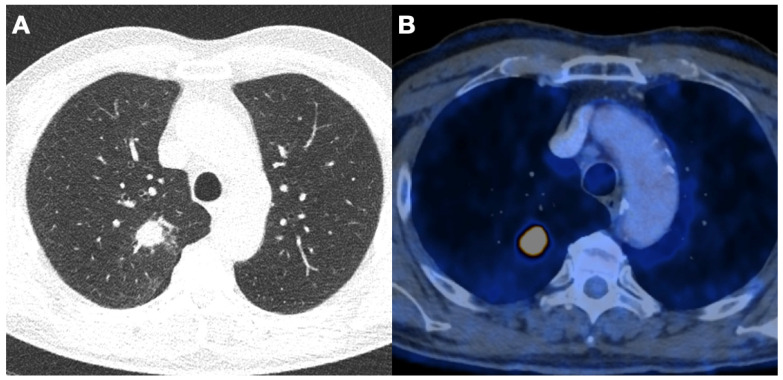
Low-dose CT (**A**) and FDG-PET/CT (**B**) images in axial plane of a 66-year-old male patient with stage IIIA squamous cell carcinoma in the upper right lobe. This previously asymptomatic patient underwent a lobectomy with systematic lymph node dissection and received adjuvant chemotherapy.

Among the screened participants, 3.6% (*n* = 4) were diagnosed with lung cancer. Diagnosis was obtained via CT guided biopsy in all cases. One male participant (68 years- old) with a PLCOm2012 of 12.2% was diagnosed with adenocarcinoma in the left lower lobe with one metastasis in the sternal manubrium (pT3, pN0, cM1b, UICC stadium IV). The participant underwent four cycles of induction chemotherapy and radiotherapy for the osseous metastasis and subsequently underwent a video-assisted thoracoscopic lobectomy and radical mediastinal lymph node dissection. This participant is without signs of metastasis or recurrence. Another male participant (66 years-old) with a PLCOm2012 of 3.5% was diagnosed with non-keratinizing squamous cell carcinoma in the right upper lobe and two mediastinal lymph node metastases (pT1, pN2, cM0, UICC stadium IIIA). Mediastinal lymph node metastasis (same side) was incidental and found only in histopathologic assessment post-surgery. The participant underwent robotic-assisted lobectomy and radical lymph node dissection followed by adjuvant chemotherapy with Cisplatin/Gemtacitabin. CT and PET/CT images are shown in Figure 2. After remission, the development of an osseous metastasis was found which is now under radiotherapy with a curative approach. Unfortunately, brain metastases were found at the follow-up scans and further steps are currently being discussed in multidisciplinary tumor boards. Another adenocarcinoma was found in a male patient (69 years-old) with a PLCOm2012 of 5.4%. The tumor was localized in the right upper lobe and no metastases were found (pT2a, pN0, cM0, UICC stadium IB). The participant underwent robotic-assisted lobectomy and radical lymph node dissection and is now without signs of further disease. One female participant was diagnosed with adenocarcinoma in the right lower lobe. Two lymph node metastases were found (pT3, pN2, cM0, UICC IIIA). Again, robotic-assisted lobectomy, radical lymph node dissection, as well as stereotactic radiotherapy, were performed. Positive lymph node status has not been described prior to the surgery and was confirmed via histopathological assessment. The participant is without signs of metastasis or recurrence. Further, 3% (*n* = 3) of participants are currently under active surveillance due to a highly suspicious nodule (Lung-RADS 4a).

### 3.5. Incidental Findings

Incidental findings were communicated to and managed by the referring physicians. No incidental findings requiring urgent attention were found. Among the participants, 71% (*n* = 80) were diagnosed with coronary sclerosis and 32% (*n* = 36) participants were found to have emphysematous parenchymal lung changes. Further, 35% of the participants (*n* = 39) were diagnosed with bronchitis. There were also pathologies found in the partially imaged upper abdomen; none of them were in need of treatment (benign liver lesions (*n* = 14), renal cysts (*n* = 11), adrenal adenomas (*n* = 2) and cholecystolithiasis (*n* = 4)). The management of incidental findings was under the responsibility of the referring physician who received the CT report.

## 4. Discussion

Lung cancer is one of the leading causes of cancer-related deaths worldwide, and early detection is crucial for improving patient outcomes [6,17]. Switzerland, like many other countries, aims to implement lung cancer screening programs to ensure the detection of lung cancers in an early, curative treatable stage. To this date, we have found four curatively treated cases of lung cancer. Another three suspicious lesions are currently under active surveillance. The recall rate in our study was 15% by combining all Lung-RADS 3 and Lung-RADS 4a lesions. We found that 81% of the participants had suspected lung nodules requiring further follow-up.

Tomonaga et al. [15] have already indicated that lung screening could be cost-effective in Switzerland, a European country with a high income and a high smoking prevalence. They estimated the cost-effectiveness of LDCT screening for lung cancer to be less than EUR 50,000 per life year gained. The economic evaluation of a health care program as the lung cancer screening relies on different aspects including the life expectancy and the quality of life potentially saved. These also take into account the productivity of each individual. In our cohort, 56% of people were active workers, which means that a potential intervention would safeguard a productive subject within society.

A screening program can only be successful if it reaches as many at-risk people as possible. In our study there is an overrepresentation of high-educated participants with 92 (82%) receiving at least post-high school training. In 2017, within the portion of the Swiss population with secondary school training, 30.1% were active smokers, whereas this percentage was 23.1% within the population with a higher educational level [14]. This might mean that we are potentially missing part of the target population, and we may need to improve recruitment strategies. On the other hand, this may be attributable to the single-center design, reaching only participants in an urban area around Zurich. Within the cohort, 73% of the participants were resident in the Zurich Canton which extends for 1729 km^2^, and the median distance travelled by each participant was 16.7 km, most of which was by public transport. These data are extremely important to verify the applicability of a future structured program in our country and to provide information on centralized vs. decentralized design. A more decentralized approach could lead to higher accessibility and therefore better representability of the Swiss population. Since CT density in Switzerland is estimated to be high, the degree of centralization could vary depending on the extent of pre-screening, screening, and evaluation. One possible approach is to decentralize diagnostics and organize reading and treatment centrally. A model for this would be the already mentioned “lung health checks” in Manchester, which were carried out with a mobile CT device [12].

Another aspect that must be evaluated is the enrollment strategy. In our population, half of the participants became aware of the pilot project through flyers, newspapers, or social media, and only 25% were invited by doctors. A previous study conducted in Switzerland demonstrated a better adherence to lung cancer diagnostic and treatment pathways with a more consistent involvement of primary care practitioner (PCPs) [18]. PCPs represent the frontline health care professionals with knowledge of patients’ general status, behavior, and clinical history, and this relationship might be important to enhance screening adherence.

Another main issue in lung cancer screening is in defining a screening protocol associated with a low recall rate and a high detection rate. In the context of screening, the recall rate is a crucial metric that reflects the proportion of individuals who are called back for additional tests or evaluations following an initial screening, often due to the detection of suspicious findings [19]. A high recall rate can lead to increased anxiety and stress among patients who are called back for further tests. This can negatively impact their well-being and quality of life, even if they ultimately receive a negative diagnosis. Rasmussen et al. [20] evaluated the psychological impact of lung screening on patients and found that receiving a false-positive result in lung cancer screening was associated with negative short-term psychosocial consequences. The ideal recall rate can vary depending on the specific screening program, the disease being screened for, and the available resources. It is often a trade-off between achieving high sensitivity (catching more cases) and maintaining an acceptable level of specificity (avoiding unnecessary recalls). In our study we evaluated lung nodules according to Lung-RADS criteria which led to a recall rate of 15%. This value is lower compared to the NLST who used a cut off-value of 4 mm nodule size which resulted in a recall rate of 27% [7]. In breast cancer screening, the Agency for Healthcare Research and Quality has recommended a target recall rate of 10% and the American Society of Radiology (ACR) has recommended a target recall rate range of 5–12% for screening mammography [21]. To date, there are no recommended cut-off values for the recall rate in lung cancer screening. Anyhow, effort should be made towards lower recall rates which do not unnecessarily put participants under emotional stress. Currently we are evaluating the emotional stress in a prospective study (BASEC-Nr. 2022-01484).

In our pilot study, we found a cancer detection rate of 3.6% which is slightly higher than in the NELSON trial (3.2%) and higher than in the NLST (2.4%) [6,7]. This may be based on the modified inclusion criteria. In addition to the NLST criteria we also used the PLCOm2012 criteria for eligibility assessment. If patients did not have “enough” pack years or did stop smoking longer than 15 years ago we still included them if they had a PLCOm2012 higher than 1.5%. Tammemägi et al. have proven the feasibility of PLCOm2012 in a lung cancer screening setting [16]. Among others, factors like ethnicity, education, BMI, family history of cancer or COPD are taken into account in the PLCOm2012 risk evaluation.

In our study three out of four patients were diagnosed with advanced, metastatic disease. However, those patients were asymptomatic at the timepoint of screening and were treated with a curative approach.

In addition to a reduction in lung cancer-specific mortality by 20%, an all-cause mortality reduction of 6.7% could be shown in the National Lung Screening Trial [22]. The effects of smoking extend beyond cancer evolution. Smokers are also at risk of premature deaths due to chronic obstructive pulmonary disease (COPD) and coronary heart disease (CHD); both conditions can also be assessed in the context of screening [23]. For subjects in the lung cancer screening window, the relative risk of death due to ischemic heart disease is greater than three times that of a non-smoker [24]. Moreover, smoking reduces the time to development of coronary artery calcium (up to 10 years earlier for current smokers) compared with non-smokers [25]. While electrocardiogram-gated CT has been the gold standard for coronary artery quantification, there is compelling evidence that in lung cancer screening programs, a non-gated CT is a robust prognostic measure of fatal or non-fatal cardiovascular events in current and former smokers [23,26]. In our study 90% of the participants were found to have coronary artery calcification, among whom 10% were rated as severe. None of them were diagnosed with cardiovascular disease prior to participation. Further, although 14% of the participants were already diagnosed with COPD prior to our study, we found emphysematous lung changes in 32%. Those ancillary findings could contribute to risk stratification as well as health management and may lead to a reduction in mortality.

Our study has several limitations. First, the cohort is small and more participants need to be recruited for evidenced-based conclusions. There is also a lack of ethnical variety among participants (all participants were white with exception of two Hispanic participants). Anyhow, the major ethnicity in the Swiss population is white and therefore an underrepresentation of other ethnicity has to be taken into account. Given the advanced stage of three out of four detected cancers, it is important to acknowledge that this factor may have implications for the cost-effectiveness of the program. This issue warrants further attention in future projects, especially those involving a larger patient cohort. Furthermore, our pilot study is based on data from only one institution. In the future, many more sites all over Switzerland have to be included to ensure a nationwide, easily accessible screening program.

## 5. Conclusions

In conclusion, preliminary results from our pilot study have shown to be effective in detecting curative treatable lung cancer and improving patient outcomes. However, challenges such as limited accessibility, a lack of awareness, and the need for standardized guidelines must be addressed to ensure the program’s long-term success. The continuous recruitment of participants and the involvement of further centers is necessary to guarantee easy access to screening programs and to continually evaluate the effectiveness of the screening program in Switzerland.

## Figures and Tables

**Table 1:** Patient demographics, socioeconomic characteristics, and risk evaluation among the screened participants.

Subject Demographic/Socioeconomic Characteristic Risk Evaluation		Results
**Demographics/Socioeconomics**		
Gender (Number, (%))		
	Male	65 (58)
	Female	47 (42)
Age (Median, (p25–75%))		61.5 (57.0–67.0)
Ethnicity (Number, (%))		
	Black	0 (0)
	Hispanic	2 (2)
	Asian	0 (0)
	White	110 (98)
	Other (included mixed race)	0 (0)
Education Level (Number, (%))		
	Less than high-school	1 (1)
	High-school	17 (15)
	Post high-school training	35 (31)
	College degree	41 (37)
	Postgraduate/Professional	18 (16)
**Risk Evaluation**		
BMI (Median, (p25–75%))		25.6 (23.3–28.0)
Smoking status (Number, (%))		
	current	85 (76)
	former	27 (24)
Pack years (Median, (p25–75%))		45 (38–57)
History of COPD, Emphysema or Chron. Bronchitis (Number, (%))		16 (14)
PCLOm 2012 (Mean (SD))		3.7 (2.5)

**Table 2:** Screening results.

Screening Results		
**LUNG-RADS**		Results
0 (Number, (%))		0 (0)
1 (Number, (%))		62 (55)
2 (Number, (%))		29 (26)
3 (Number, (%))		14 (13)
4a (Number, (%))		3 (3)
4b (Number, (%))		4 (4)
Carcinomas (Number, (%))		4 (4)
	Adenocarcinoma	3 (3)
	Squamous cell carcinoma	1 (1)
**Incidental Findings**		
Coronary Sclerosis (Number, (%))		
	non	32 (29)
	mild	38 (34)
	moderate	32 (29)
	severe	10 (10)
Emphysema (Number, (%))		36 (32)

## Data Availability

The data presented in this study are available on request from the corresponding author. The data are not publicly available due to privacy.
